# Translating, adapting, and validating the medical student version of the patient care ownership scale for use in Japan

**DOI:** 10.1186/s12909-024-05704-8

**Published:** 2024-06-28

**Authors:** Hirohisa Fujikawa, Mikio Hayashi, Daisuke Son, Kayo Kondo, Masato Eto

**Affiliations:** 1 Center for General Medicine Education, School of Medicine, Keio University, 35 Shinanomachi, Shinjuku-ku, Tokyo, 160-8582 Japan; 2 Department of Medical Education Studies, International Research Center for Medical Education, Graduate School of Medicine, The University of Tokyo, Bunkyo-ku, Tokyo, Japan; 3 Center for Health Professions Education, Kansai Medical University, Hirakata, Osaka Japan; 4 Master of Medical Sciences in Medical Education, Harvard Medical School, Boston, MA USA; 5 Department of Community-based Family Medicine, Faculty of Medicine, Tottori University, Yonago, Tottori Japan; 6 School of Modern Languages and Cultures, Durham University, Durham, UK

**Keywords:** Patient care ownership, Medical students, Reliability, Validity, Factor analysis

## Abstract

**Background:**

Patient care ownership (PCO) among medical students is a growing area in the field of medical education. While PCO has received increasing attention, there are no instruments to assess PCO in the context of Japanese undergraduate medical education. This study aimed to translate, culturally adapt, and validate the PCO Scale – Medical students (PCOS-S) in the Japanese context.

**Methods:**

We collected survey data from fifth- and sixth-grade medical students from five different universities varying in location and type. Structural validity, convergent validity, and internal consistency reliability were examined.

**Results:**

Data from 122 respondents were analyzed. Factor analysis of the Japanese PCOS-S revealed three factors with Cronbach’s alpha values exceeding the satisfactory criterion (0.70). A positive correlation was observed between the total Japanese PCOS-S scores and the global rating scores for the clinical department as a learning environment (Pearson’s correlation coefficient = 0.61).

**Conclusions:**

We conducted the translation of the PCOS-S into Japanese and assessed its psychometric properties. The Japanese version has good reliability and validity. This instrument has potential value in assessing the development of medical students’ PCO.

## Background

Medical professionalism is an important competency for physicians and the cornerstone that underpins the quality medical education and clinical practice [1]. It articulates the core values of physicians and establishes the rules and norms that guide physicians’ behavior [2]. There is a consensus that national and international professional associations have recognized the importance of medical professionalism and defined a framework for its core elements [3, 4]. Therefore, it remains a powerful force in the medical education community.

Patient care ownership (PCO), affective-cognitive state in which physicians use emotional and intellectual skills to make decisions in clinical practice, is an essential component of medical professionalism [5]. PCO was previously described as “the philosophy that one knows everything about one’s patients and does everything for them [6].” More recently, the concept has gradually changed against the backdrop of international working hour regulations for physicians and the promotion of interprofessional work and team-based patient care. For example, a qualitative study in the U.S. revealed that PCO was composed of the following components: advocacy for the patient, communication and care coordination, decision-making, follow-up in completing tasks of patient care, knowledge, leadership, attitudes of doing more than the minimum required, responsibility, serving as primary or main care provider, demonstrating initiative, and providing the best care [7]. Another qualitative study identified three key components of PCO in the night float system of a Canadian internal medicine residency program: continuous personal concern for patients, autonomous decision-making, and knowledge of patients’ problems [8]. While PCO is an important element that should be mastered during residency [9], it should also be cultivated during medical school, when medical students begin to form professional identities [10, 11].

There have been many studies on PCO, most of which focused on medical residents [12]. Most of these studies employ qualitative research design. In addition to the aforementioned studies investigating the components of PCO, Robinson et al. investigated the relationship between PCO and pediatric residents’ decision-making opportunities [13]. The PCO Scale (PCOS) was developed for U.S. medical residents by Djulbegovic et al. in 2019 [14, 15] and its Japanese translation by Fujikawa et al. in 2021 [16]. Since then, quantitative studies on PCO among medical residents have gradually been conducted. For example, a study conducted in Japan explored the association between PCO and personal or environmental factors among medical residents using the Japanese medical residents’ version of the PCOS [17].

Conversely, few studies have focused on PCO among medical students because there were no instruments to measure PCO among medical students. Recently, a version of the PCOS for medical students (PCOS-S) was developed and validated by Wyatt et al. in 2023 [12]. Medical undergraduates are expected to take patient ownership appropriate to their level of training; since Djulbegovic’s scale is too advanced for medical undergraduates, Wyatt et al. created a scale adapted for them, PCOS-S. There is no corresponding tool in Japan. Developing such a tool would be useful to conduct future international studies and to track the development of medical students in important aspects of becoming a physician. Therefore, the aim of this study was to construct the Japanese version of the PCOS-S by translating, adapting and validating the scale for use in Japan.

## Methods

### Study design and participants

This multicenter cross-sectional study was conducted at five universities in Japan between June and July 2023. The participating universities varied in location and type to ensure the diversity of the student population. Eligible participants were all fifth- and sixth-grade medical students (i.e., clinical-year medical students) at the five universities. Anonymous online questionnaires, using a weblink created by SurveyMonkey (www.surveymonkey.com), were distributed to all the eligible participants by the director of undergraduate medical education at each university. Since the questionnaire was designed to be applicable only to inpatient care at the time of survey response, we decided to include only students who were in charge of inpatients at the time of the study.

### Curriculum of medical undergraduates in Japan

Herein, we briefly describe the curriculum of medical students in Japan to aid understanding the study context. The Japanese undergraduate medical education is six years long [18, 19]. It typically consists of four years of preclinical education and two years of clinical education. A shift in clinical clerkship from the traditional “observation” model to the “participation” model has been continually advocated since the 2000s. In reality, however, the shift has made little progress [20]. In addition, although the Model Core Curriculum for Medical Education, developed by the Ministry of Education, Culture, Sports, Science and Technology, serves as the guideline for curricula of all medical schools [21], actual medical education varies from university to university and department to department.

### Measures

The questionnaire consisted of three parts:1) informed consent, 2) demographic questions, and 3) questions PCO.

1) Informed consent.

Informed consentwas obtained from all the participants. We asked the participants to tick a consent box to indicate their agreement to take part in the survey.

2) Demographic questions.

In the second part of the questionnaire, we collected data on participants’ demographic information, including gender, name of the university, grade, and clinical department to which they were assigned to at the time of the survey. We also asked them whether the participants were in charge of inpatients in the clinical clerkship at the time of the study because the questionnaire was only used in inpatient care settings. In addition, we asked the participants to what extent the department was educational (as described in detail hereafter).

3) Questions regarding PCO.

The third part of the questionnaire asked the participants about their experiences with PCO. The PCOS-S used in this study includes 19 items [12]. Each item is answered on a six-point Likert scale (from 1 = strongly disagree to 6 = strongly agree). Scale scores are calculated by simple summation of the items, with higher scores indicating higher PCO. Factor analysis supported a four-factor structure: Factor 1 (seven items, *team inclusion*), Factor 2 (five items, *accountability*), Factor 3 (four items, *territoriality*), and Factor 4 (three items, *self-confidence*) [12].

The Japanese version of the scale was developed using an established translation process [22] as follows: We obtained permission from the original author for the translation of the scale into Japanese. Second, three translators (HF, DS, and KK) performed forward translations from English to Japanese. They had experience translating survey instruments [16, 23, 24], including medical residents’ version of the PCOS [16]. Third, the three translations were reviewed and synthesized by three translators (Version 1). Fourth, Version 1 was back translated from Japanese into English by professional bilingual translators who were not involved in the study. Three translators compared the above two versions, the back-translated version with the original, and revised them to create Version 2. Fifth, FH emailed the original author, asked for a review, and the author confirmed that there was no need for correction of the translation. Sixth, a medical education expert (MH) reviewed Version 2 and found no particular problems with the translation. Seventh, we conducted a pilot survey with three medical students to check if there were any difficulties in understanding the content and completing the questionnaire. Pilot tests revealed no major problematic items. Finally, all authors confirmed the instrument’s face and content validity. Therefore, Version 2 was considered acceptable for use in collecting data.

### Statistical analysis

The following four steps were taken to validate the Japanese version of the PCOS-S:

First, we performed an exploratory factor analysis (EFA) to test the structural validity of the scale. After checking the Kaiser–Meyer–Olkin (KMO) measure of sampling adequacy and Bartlett’s sphericity test, we conducted an EFA. The recommended criteria for reliable EFA are as follows: a KMO value of 0.60 or greater and a significant Bartlett’s test of sphericity [25]. We used promax rotation in conjunction with the maximum likelihood estimation. A parallel analysis was conducted for factor extraction [26]. Items with factor loading below 0.40 were excluded.

Second, convergent validity was assessed through hypothesis testing. A previous study showed a link between PCO and the level of the clinical department as a learning environment among medical residents [17]. In this study, the association between the Japanese version of the PCOS-S and global rating scores of the clinical department as a learning environment was assessed using Pearson’s correlation coefficients. Consistent with a previous study [17], the question asking to rate the clinical department as a learning environment was as follows: “Using any number from 0 to 10, where 0 is the worst possible and 10 is the best possible, what number would you use to rate your current department as a learning environment?” Pearson correlation coefficients above 0.30 were considered meaningful [27].

Third, the internal consistency reliability was assessed using Cronbach’s alpha. Previous studies suggest that a value of 0.70 or higher is the satisfactory criterion [28].

Fourth, descriptive statistics were calculated on the scale scores, including the mean, standard deviation, and observed range. We also conducted an independent t-test to investigate the possible influence of participant’s year group on the PCOS-S scores. We chose a complete case analysis. R version 4.3.1 (R Foundation for Statistical Computing, Vienna, Austria; www.R-project.org) was used for the data analysis. To conduct the EFA, we used psych version 2.3.6 and GPArotation version 2023.8-1 [29, 30].

### Ethical considerations

The participants were enrolled in a drawing for one of five ¥5,000 vouchers. Ethical approval was obtained from the Institutional Review Board of the University of Tokyo (2022066NI).

## Results

**Fig. 1:**
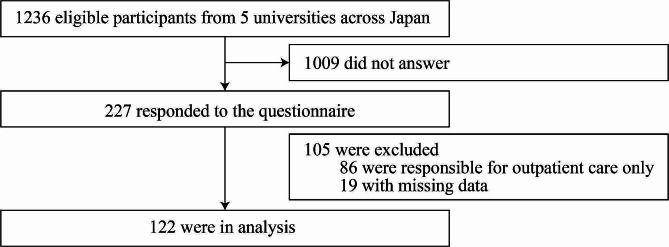
Participants flowchart

A flowchart of the participants is shown in Fig. 1. Of the total 1236 eligible participants, 227 responded to the online questionnaire. Of these, 86 students who were responsible for outpatient care only and 19 students who were responsible for inpatient care but had missing data were excluded. Therefore, 122 students’ responses were included in the final analysis. The characteristics of those participants are presented in Table 1. Responses to each item on the scale are shown in Table 2.

**Table 1:** Characteristics of the participants (*N* = 122)

	*N* (%)
Gender	
Male	86 (70.5)
Female	35 (28.7)
Non-binary	1 (0.8)
University type	
National	80 (65.6)
Private	42 (34.4)
Year	
Fifth	80 (65.6)
Sixth	42 (34.4)
Clinical department	
Internal medicine	53 (43.4)
Surgery	23 (18.9)
Psychiatry	10 (8.2)
Pediatrics	9 (7.4)
Obstetrics and Gynecology	9 (7.4)
Orthopedics	6 (4.9)
Dermatology	3 (2.5)
Emergency medicine	2 (1.6)
Neurosurgery	2 (1.6)
Urology	2 (1.6)
Others	3 (2.5)

**Table 2:** Responses to the Japanese version of the PCOS-S items (*N* = 122): number (%)

Items (as in original English version)	1	2	3	4	5	6
Q1. I have a sense of belonging in this healthcare team	4 (3.3)	14 (11.5)	18 (14.8)	54 (44.3)	25 (20.5)	7 (5.7)
Q2. I have close bonds with my healthcare team	4 (3.3)	17 (13.9)	19 (15.6)	52 (42.6)	25 (20.5)	5 (4.1)
Q3. I feel like I am a member of the healthcare team	3 (2.5)	16 (13.1)	24 (19.7)	50 (41.0)	25 (20.5)	4 (3.3)
Q4. I am an active member of the healthcare team	10 (8.2)	26 (21.3)	20 (16.4)	49 (40.2)	15 (12.3)	2 (1.6)
Q5. Being a member of this healthcare team is important to me	9 (7.4)	16 (13.1)	21 (17.2)	41 (33.6)	26 (21.3)	9 (7.4)
Q6. I feel included on this team	4 (3.3)	16 (13.1)	24 (19.7)	42 (34.4)	27 (22.1)	9 (7.4)
Q7. I enjoy working as a team	0 (0)	8 (6.6)	11 (9.0)	45 (36.9)	39 (32.0)	19 (15.6)
Q8. I have the right to hold others accountable for the care of my patient	11 (9.0)	16 (13.1)	22 (18.0)	43 (35.2)	24 (19.7)	6 (4.9)
Q9. I have the right to ask for others to justify their management decisions when it pertains to my patients	3 (2.5)	8 (6.6)	8 (6.6)	48 (39.3)	40 (32.8)	15 (12.3)
Q10. I consistently hold myself accountable for my patients’ care	14 (11.5)	17 (13.9)	25 (20.5)	44 (36.1)	14 (11.5)	8 (6.6)
Q11. I have a right to know what is going on with my patient.	1 (0.8)	1 (0.8)	3 (2.5)	22 (18.0)	54 (44.3)	41 (33.6)
Q12. When I make plans for patient care, I am certain I can make them work	5 (4.1)	15 (12.3)	50 (41.0)	42 (34.4)	7 (5.7)	3 (2.5)
Q13. I feel I need to protect my work (i.e. notes, slides, records) from others on my healthcare team.*	20 (16.4)	21 (17.2)	32 (26.2)	25 (20.5)	20 (16.4)	4 (3.3)
Q14. I feel I need to protect my ideas about patients from my peers.*	4 (3.3)	15 (12.3)	44 (36.1)	31 (25.4)	23 (18.9)	5 (4.1)
Q15. I feel that people I work with should not invade my areas of responsibility.*	1 (0.8)	4 (3.3)	16 (13.1)	44 (36.1)	39 (32.0)	18 (14.8)
Q16. I feel I have to assert my role on this healthcare team.*	2 (1.6)	11 (9.0)	46 (37.7)	35 (28.7)	22 (18.0)	6 (4.9)
Q17. I feel insecure about my ability to do things related to patient care.*	11 (9.0)	42 (34.4)	46 (37.7)	15 (12.3)	8 (6.6)	0 (0)
Q18. I do not feel sufficiently confident to try different ways of providing patient care.*	15 (12.3)	39 (32.0)	43 (35.2)	19 (15.6)	5 (4.1)	1 (0.8)
Q19. I have difficulty using my knowledge in patient care on this healthcare team.*	5 (4.1)	14 (11.5)	40 (32.8)	41 (33.6)	18 (14.8)	4 (3.3)

*These were reverse items. Therefore, these items were reverse-coded (e.g., 1 was changed to 6, 2 was changed to 5)

*Abbreviations PCOS-S* Patient Care Ownership Scale – Medical students version, *Q* Question

1) Structural validity.

The KMO value was 0.80, exceeding the required 0.60, and the result of Bartlett’s test of sphericity was χ^2^ = 1170.557 (df = 171, *p* < 0.001). As these results indicated that the items of the scale were suitable for EFA, we decided to conduct it.

After several iterations, the EFA suggested that 6 of the 19 items should be deleted because of low factor loadings (< 0.37). The authors reviewed six items and concluded that they should be deleted because they did not appear to fit the Japanese context. The final results of the EFA are listed in Table 3. The results suggest a three-factor structure, and all factor loadings presented good values (> 0.40).

Through discussions among the authors, the following three factors were identified: Factor 1 (7 items), team engagement; Factor 2 (3 items), self-confidence; and Factor 3 (3 items), territoriality.

**Table 3:** The final results obtained from exploratory factor analysis of the 13-item Japanese version of the PCOS-S (*N* = 122)

Items (as in original English version)	Factor loading		
	F1	F2	F3
Q3. I feel like I am a member of the healthcare team	0.964		
Q2. I have close bonds with my healthcare team	0.925		
Q1. I have a sense of belonging in this healthcare team	0.903		
Q6. I feel included on this team	0.862		
Q4. I am an active member of the healthcare team	0.717		
Q5. Being a member of this healthcare team is important to me	0.599		
Q10. I consistently hold myself accountable for my patients’ care	0.418		
Q18. I do not feel sufficiently confident to try different ways of providing patient care.*		0.949	
Q17. I feel insecure about my ability to do things related to patient care.*		0.878	
Q19. I have difficulty using my knowledge in patient care on this healthcare team.*		0.441	
Q14. I feel I need to protect my ideas about patients from my peers.*			0.994
Q13. I feel I need to protect my work (i.e. notes, slides, records) from others on my healthcare team.*			0.689
Q15. I feel that people I work with should not invade my areas of responsibility.*			0.539
	**Value**		
**Eigenvalue**	4.74	1.16	0.91
**% of variance explained**	34.1	14.3	13.6

*These were reverse items

*Abbreviations F* Factor, *PCOS-S* Patient Care Ownership Scale – Medical students version, *Q* Question

2) Convergent validity.

We examined the Pearson correlation coefficient between the Japanese PCOS-S scores and the global rating scores of the clinical department as a learning environment. The value was 0.61, which exceeded the required 0.30 (*p* < 0.001).

3) Internal consistency reliability and descriptive statistics.

Table 4 shows the internal consistency reliability and descriptive statistics of our scale. The overall Cronbach’s alpha for the scale was 0.81. The Cronbach’s alpha values of all the factors exceeded the satisfactory criterion of 0.70. Table 5 shows the result of the independent t-test to examine the influence of participants’ year group on the PCOS-S scores. 6th-year students had significantly higher PCO than 5th-year students. Thus, the final version of the Japanese PCOS-S was developed.

**Table 4:** Descriptive features of the Japanese version of the PCOS-S

Domain	Number of items	Mean	Standard deviation	Observed range	Cronbach’s alpha
Total	13	45.6	8.5	20–73	0.81
Team engagement	7	25.6	6.8	7–42	0.90
Self-confidence	3	9.0	2.7	3–16	0.79
Territoriality	3	11.1	3.0	3–18	0.75

*Abbreviations PCOS-S* Patient Care Ownership Scale – Medical students version

**Table 5:** The mean difference of the Japanese version of the PCOS-S

	Difference in score	95% CI	*p* value
5th-year	Reference category		
6th-year	5.82	2.77 to 8.86	< 0.001

## Discussion

We developed a 13-item Japanese version of the PCOS-S to assess PCO in medical students and examined its psychometric properties. PCO assessment plays a vital role in medical education. The instrument developed in this study can improve the quality of undergraduate medical education and PCO research.

In the internal consistency reliability analysis, Cronbach’s alpha for all subscales met the satisfactory criterion. This finding was consistent with that of the original study. The original scale had four subscales with Cronbach’s alpha coefficients of 0.78 or higher. Thus, the scales appeared to have adequate internal consistency and reliability.

In the present study, the factor analysis showed a three-factor structure, whereas the original scale of the English version has a four-factor structure. Comparing the Japanese and English versions, most items in the accountability dimension were excluded from the former. There may be two possible reasons. First, differences in the content of medical education between Japan and the U.S. may have an impact on the results. As we described in the Methods, the clinical clerkship in Japan is often conducted in an “observation” model [20]. Accordingly, Japanese medical students may have few experiences of accountability in each clinical clerkship. Second, it is possible that this discrepancy may be a reflection of the unique characteristics of Japanese culture. It is often said that Japanese society is traditionally ill-suited to the concept of accountability [31]. In Japan, the Western notion of “accountability” was first introduced in the mid-1990s [32]. It was a difficult concept to understand in Japanese society and is currently often translated as “*setsumei sekinin*,” which literally means “duty to explain” [32]. However, it has been pointed out that by translating the term accountability into “*setsumei sekinin*,” the original broad meaning of accountability has been lost in Japan. For example, “*setsumei sekinin*” does not include responsibility for results, responsibility to have other people explained, or the ability to explain and gain acceptance [31]. Behind this is a part of the Japanese culture that favors stillness and silence. In Japan, silence is important because of Zen Buddhism [33–35]. The discourse on silence has been found not only in traditional literature but also in modern Zen practices, in which silence is understood as an expressive form of understanding [34]. In other words, the culture values refraining from speaking up and questioning in public and avoiding people who do so [31]. This long-standing attitudinal difference could explain why most items in the accountability dimension disappeared in our Japanese version, resulting in a three-factor structure. There would be a need for future research to confirm the factor structure.

Our scale is the first validated instrument to assess PCO in a Japanese undergraduate medical education setting. Factor analysis appears to have succeeded in creating a scale that is commensurate with the current state of clinical clerkship for Japanese medical students, as described earlier. In addition, in our sample, 6th graders had significantly higher PCO than 5th graders, suggesting that educational intervention may nurture PCO. We suggest that medical educators in Japan use our scale to assess the PCO of medical students, which we believe will be an effective tool to guide to quality improvements in undergraduate medical education. It would be useful to observe how the PCO of medical students changes over time during their clerkships. We also recommend that researchers use our instrument to examine the association between the PCO of medical students and their clinical outcomes (e.g., patient experience), which has not yet been explored. Moreover, developing a PCOS-S in languages other than English and Japanese would facilitate international research and enrich medical students’ PCO knowledge.

We should note a couple of potential limitations. First, the response rate and sample size were relatively low. Including more medical students in future studies would strengthen our argument for using this scale. Second, we cannot exclude the possibility of selection bias. It is possible that only medical students with high PCO responded to the questionnaire. Third, as the questionnaire was for a particular clinical department inpatient clerkship at the time of response, the educational curriculum of the department would influence the result. Fourth, we did not carry out confirmatory factor analysis to verify the factor structure resulting from the EFA. Future studies should test the three-factor structure using confirmatory factor analysis. Fifth, validity other than structural and convergent validity (e.g., discriminant validity) and reliability other than internal consistency reliability (e.g., test-retest reliability) were not evaluated. Further research is required to assess these psychometric properties. Sixth, because this was a scale validation study, the association between PCOS-S overall and subscale scores and other concepts is unknown. Future research would deepen our knowledge of PCOS-S, for example, by investigating the relationship between PCOS-S scores and academic performance.

## Conclusions

We translated the PCOS-S into Japanese, and tested and verified its structural validity, convergent validity, and internal consistency reliability. The Japanese PCOS-S has good reliability and validity. Three factors were extracted from the factor analysis. This instrument could be useful for quality improvement and research on bridging undergraduate medical education for PCO to clinical settings and practices. Further studies are required to confirm the robustness of this scale.

## Data Availability

The datasets generated and analyzed in this study can be made available by the corresponding author upon reasonable request.

## Abbreviations

EFA: Exploratory factor analysis
KMO: Kaiser–Meyer–Olkin
PCO: Patient care ownership
PCOS: Patient care ownership scale
PCOS-S: Patient care ownership scale – Medical students version
